# Long-term Follow Up of a Boy with Unilateral Autosomal Dominant Polycystic Kidney Disease and Contralateral Renal Agenesis

**DOI:** 10.34763/devperiodmed.20172104.380383

**Published:** 2018-01-02

**Authors:** Grażyna Krzemień, Agnieszka Turczyn, Małgorzata Pańczyk-Tomaszewska, Aleksandra Jakimów-Kostrzewa, Agnieszka Szmigielska

**Affiliations:** 1 Department of Pediatrics and Nephrology, Medical University of Warsaw, Warsaw Poland; 2 Department of Pediatric Radiology, Medical University of Warsaw, Warsaw Poland

**Keywords:** autosomal dominant polycystic kidney disease, ADPKD, renal agenesis, chronic renal disease, hypertension, children, torbielowatość nerek typu dominującego, ADPKD, agenezja nerki, przewlekła choroba nerek, nadciśnienie, dzieci

## Abstract

In patients with autosomal dominant polycystic kidney disease (ADPKD) coexisting abnormalities of the urinary tract are considered rare. Only a few patients with ADPKD and congenital abnormalities of the kidney and urinary tract- renal agenesis, hypoplasia, aplasia, horseshoe kidney, ectopic multicystic dysplasic kidney, or subpelvic junction obstruction were reported. Renal agenesis occurs in approximately 1 in 1.500.000-3.000.000 patients with ADPKD. We report a boy with ADPKD and renal agenesis diagnosed at the age of 12 years. ADPKD was diagnosed in some other members of the family. Additionally to kidney changes, mitral valve prolapse was found on echocardiography. At the age of 18 years high normal blood pressure was recognized and laboratory tests demonstrated: serum creatinine 1.0 mg/dl, glomerular filtrate rate 97.9 ml/min/1.73m^2^, isotopic creatinine clearance (Tc-99mDTPA) 99 ml/min/1.73m^2^, normal urinalysis, no microalbuminuria.

**Conclusions:**

In children with positive family history of ADPKD, screening ultrasonography of the kidney performed at the request of the family, allows the early diagnosis of sporadic present abnormalities of the kidney and urinary tract.

## Streszczenie

U pacjentów z torbielowatością nerek typu dominującego (ADPKD) wady układu moczowego występują stosunkowo rzadko. Tylko u nielicznych pacjentów stwierdza się inne wrodzone wady nerek i układu moczowego takie jak agenezja, hipoplazja, aplazja, nerka podkowiasta, ekotopowa nerka dysplastyczna wielotorbielowata lub podmiedniczkowe zwężenie moczowodu. Częstość występowania agenezji nerek u osób z ADPKD określa się na 1 in 1.500.000-3.000.000. Przedstawiamy przypadek chłopca z ADPKD i agenezją nerki rozpoznaną w 12 roku życia. Oprócz zmian w nerkach, w echokardiografii rozpoznano wypadanie płatka zastawki mitralnej. ADPKD rozpoznano również u innych członków rodziny. W wieku 18 lat chłopiec miał wysokie prawidłowe ciśnienie tętnicze krwi, stężenie kreatyniny we krwi 1,0mg/dl, GFR - 97.9 ml/min/1.73m^2^, izotopowy klirens kreatyniny (Tc-99mDTPA) - 99 ml/min/1.73m^2^, prawidłowe badanie moczu, nie stwierdzono mikroalbuminurii.

**Wnioski:**

U dzieci z wywiadem rodzinnym w kierunku ADPKD, przesiewowe badanie ultrasonograficzne jamy brzusznej wykonane na prośbę rodziny pozwala wykryć rzadko współistniejące wady nerek i układu moczowego.

## Introduction

Autosomal dominant polycystic kidney disease (ADPKD) is the most common inherited kidney disease [1]. The prevalence of ADPKD is 1 per 400-1000 live births [2]. Mutations in two genes are responsible for this disease: PKD1 (chromosome 16p13.3) and PKD2 (4q21). They encode proteins polycystin-1 and polycystin-2, respectively [3]. Mutations in the PKD1 account for 80% to 90% of the cases and in the PKD2 for the remaining ones [2, 4, 5]. Mutations of PKD genes lead to uncontrolled proliferation of tubule cells and the development of fluidfilled cysts. Renal cystogenesis results in enlargement of the kidneys, inflammation of parenchyma and secondary fibrosis of parenchyma [6, 7].

The incidence of unilateral renal agenesis is 1 per 500-2000 live births [8]. This malformation results from a development failure of the ureteric bud and the metanephric mesenchyme. Renal agenesis can be associated with mutations of genes involved in the induction of the ureteric bud. It can be an isolated abnormality or part of genetic, multiorgan defects [9]. Renal agenesis occurs in approximately 1 in 1.500.000-3.000.000 patients with ADPKD [10]. For the first time in the literature we report a child with ADPKD and unilateral renal agenesis.

## Case report

**Fig. 1:**
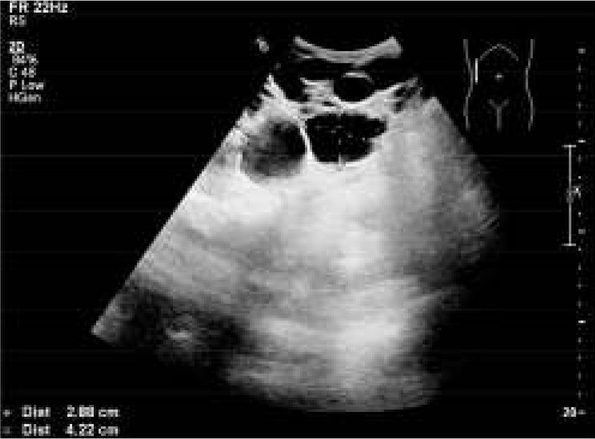
Ultrasonography shows right kidney with many cysts up to 42 mm in diameter.

The boy was born at term, from the first pregnancy and delivery with a birth weight of 3800g, and 10 points on the Apgar score. The perinatal period was normal. The family history revealed ADPKD in the grandfather and the grandfather’s sister, who both died because of end-stage renal disease (ESRD) at the age of 62 and 65 years, respectively. ADPKD was also diagnosed in the child’s mother at the age of 18 years. At the age of 6 years, the patient was diagnosed with a murmur in the heart. Echocardiography was performed and it showed mitral valve prolapse. The electrocardiogram (ECG) and chest X-ray were normal. Ultrasonography (US) of the abdomen was performed for the first time at the age of 12 years, at the request of the mother. US revealed left renal agenesis, hypertrophy of the right kidney with 129 mm in diameter and two cysts 5-6mm in diameter. On the US pyelocalyceal system, the bladder, liver, spleen, and pancreas were normal. The child was admitted to the Nephrology Department for diagnosis and treatment. Physical examination revealed no abnormalities, blood pressure (BP) and ophtalmoscopic examination were normal. Normal kidney function was found based on laboratory tests: urea-27 mg/dl (normal range 17-45 mg/dl), creatinine-0.6 mg/dl (normal range 0.2-0.7 mg/dl), glomerular filtration rate (GFR) was 109 ml/min/1.73m^2^ (normal range 90-120 ml/min/1.73m^2^), urinalysis was normal. Static scintigraphy (DMSA) showed left renal agenesis, and hypertrophy of the right kidney with normal function. Voiding cystourethography ruled out vesicoureteral reflux. The boy was followed up in an outpatient clinic. At the age of 18 years, his physical development was normal: height 178 cm (50-75 percentile), weight 72 kg (75-90 percentile), BP 125-130/65-70 mmHg, in ambulatory pressure monitoring (ABPM) mean systolic (SBP) and diastolic (DBP) blood pressure was on the 50th percentile for sex, and height, BP load and BP circadian profile were normal, with overnight drop of BP, with MAP 10%, SBP 13%, DBP 15% (normal value ≥ 10% ). Laboratory tests were as follows: urea-25.6 mg/dl, creatinine-1.0 mg/dl, GFR 97.9 ml/min/1,73m^2^, isotopic creatinine clearance (Tc-99mDTPA) 99 ml/min/1.73m^2^, normal urinalysis, albuminuria below 20 mg/a day. US of the abdomen showed hypertrophy of the right kidney with 138 mm in diameter and many cysts up to 40 mm in diameter. Liver, spleen and pancreas were normal (fig. 1).

The boy was born at term, from the first pregnancy and delivery with a birth weight of 3800g, and 10 points on the Apgar score. The perinatal period was normal. The family history revealed ADPKD in the grandfather and the grandfather’s sister, who both died because of end-stage renal disease (ESRD) at the age of 62 and 65 years, respectively. ADPKD was also diagnosed in the child’s mother at the age of 18 years. At the age of 6 years, the patient was diagnosed with a murmur in the heart. Echocardiography was performed and it showed mitral valve prolapse. The electrocardiogram (ECG) and chest X-ray were normal. Ultrasonography (US) of the abdomen was performed for the first time at the age of 12 years, at the request of the mother. US revealed left renal agenesis, hypertrophy of the right kidney with 129 mm in diameter and two cysts 5-6mm in diameter. On the US pyelocalyceal system, the bladder, liver, spleen, and pancreas were normal. The child was admitted to the Nephrology Department for diagnosis and treatment. Physical examination revealed no abnormalities, blood pressure (BP) and ophtalmoscopic examination were normal. Normal kidney function was found based on laboratory tests: urea-27 mg/dl (normal range 17-45 mg/dl), creatinine-0.6 mg/dl (normal range 0.2-0.7 mg/dl), glomerular filtration rate (GFR) was 109 ml/min/1.73m^2^ (normal range 90-120 ml/min/1.73m^2^), urinalysis was normal. Static scintigraphy (DMSA) showed left renal agenesis, and hypertrophy of the right kidney with normal function. Voiding cystourethography ruled out vesicoureteral reflux. The boy was followed up in an outpatient clinic. At the age of 18 years, his physical development was normal: height 178 cm (50-75 percentile), weight 72 kg (75-90 percentile), BP 125-130/65-70 mmHg, in ambulatory pressure monitoring (ABPM) mean systolic (SBP) and diastolic (DBP) blood pressure was on the 50th percentile for sex, and height, BP load and BP circadian profile were normal, with overnight drop of BP, with MAP 10%, SBP 13%, DBP 15% (normal value ≥ 10% ). Laboratory tests were as follows: urea-25.6 mg/dl, creatinine-1.0 mg/dl, GFR 97.9 ml/min/1,73m^2^, isotopic creatinine clearance (Tc-99mDTPA) 99 ml/min/1.73m^2^, normal urinalysis, albuminuria below 20 mg/a day. US of the abdomen showed hypertrophy of the right kidney with 138 mm in diameter and many cysts up to 40 mm in diameter. Liver, spleen and pancreas were normal (fig. 1).

## Discussion

The diagnosis of ADPKD is based on family history and imaging findings – renal cysts in ultrasonography [6, 7]. There is no established age limit for the first US, but in persons with a family history of ADPKD, it is usually performed between 20 and 30 years of age [7, 11, 12]. Available data do not recommend screening imaging tests in pediatric patients at risk, most of all because of the diffculty in confirming adequate diagnosis and lack of proven treatment [13, 14, 15]. Positive diagnosis can also have a negative influence on children’s personal life, education and career [12, 16]. The decision to perform US of the abdomen should be made together with the parents or guardian according to the clinical status and pros and cons of such a test. [11, 16]. Some authors recommended routine US *in utero* and postnatally, because about 1-2% children develop ADPKD very early [2, 3, 17]. According to Rahbari-Oskoui [4], in children between 0-15 years with family ADPKD, the presence of one cyst and/or enlarged hyperechogenic kidneys is highly suggestive of the disease. A genetic test is not a standard procedure in clinical practice, but it is mainly performed on potential living related kidney donors under the age of 40, with normal ultrasound of the kidneys and ADPKD in the family [2, 5, 15]. In some cases genetic testing is performed in very early-onset cases diagnosed in children under 2 years of age and in patients with nonspecific changes in USG and negative family history [5, 18]. *De-novo* mutation appears in approximately 3-15% patients with ADPKD [2, 3, 14, 17].

Clinical signs of ADPKD usually occur in the 3rd-4th decade of life, but can be diagnosed earlier. Abdominal pain, lumbar pain, hypertension, haematuria, proteinuria, urinary tract infections, urolithiasis and liver cysts present in children [1, 11]. Less common signs are other organ cysts, intra-cranial aneurysms and mitral valve prolapse [11, 19]. In the very early cases of disease oligohydramnios, hypertension and chronic kidney disease (CKD) are quite common [3, 11]. Family history of ADPKD was positive in the boy reported. Ultrasound of the abdomen was performed at the age of 12 years, at the request of the mother, and revealed two cysts in one kidney and agenesis of the contralateral kidney. At the time of the diagnosis, laboratory tests were normal and there were no clinical signs of the disease. High normal BP was found at the age of 18 years [20, 21]. Serum creatinine, GFR, isotopic creatinine clearance and urinalysis were normal, microalbuminuria was not present.

Abnormalities in the urinary tract in patients with ADPKD are very rare. Since 1974, only a few adult patients with ADPKD and unilateral renal agenesis, hypoplasia or aplasia [10, 22, 23, 24, 25, 26], ectopic multicystic dysplastic kidney [27], as well as subpelvic junction obstruction [28, 29, 30] were reported. More than 20 patients with ADPKD and horseshoe kidney were described [31, 32, 33]. In patients with renal agenesis, hypertrophy of the solitary kidney associated with hyperfiltration is observed. The long-term consequences of hyperfiltration can be sclerosis of the glomerulus, a decreased number of nephrons and interstitial fibrosis. It can lead to CKD and ESRD. Patients with a solitary functioning kidney and additional abnormalities of the urinary tract can develop early hypertension, proteinuria and CKD [8, 9]. Among the 8 adult patients with ADPKD and unilateral renal agenesis that were reported, one developed ESRD at the age of 34 and 4 patients at the age of 45-66 years. GFR in three patients at the age between 23-40 years was 59-83 ml/min/1.73m^2^ [10].

The increased number of cysts and their enlargement correlate with the progression of CKD [7]. Progression to ESRD is much faster in patients with PKD1 mutation than with PKD2 [5, 11]. Hypertension occurs in 6-35% of the children and is the most important modifiable risk factor of CKD progression in ADPKD patients [11, 19]. That is why screening for hypertension in children with a family history of ADPKD should be part of medical evaluation and be performed from the age of 5 years and followed up every 3 years if no hypertension is found [12, 17]. All patients with hypertension, proteinuria or microalbuminuria should receive an angiotensin-converting enzyme inhibitor (ACEI) or angiotensin receptor blocker (ARB) therapy [11, 19]. Additionally, a recommended therapy is an abundant supply of low-sodium fluid, which inhibits the secretion of ADH and collection of fluid inside cysts [12], as well as reduces BP and albuminuria in patients with CKD [16]. Evidence is limited and unclear whether renoprotective agents are e+ective for inhibiting progression of the disease [12, 17].

The boy with ADPKD and unilateral renal agenesis under discussion has a higher risk to develop ESRD. The reason for the faster progression to ESRD may be his solitary kidney as well as high normal BP and large cysts in the kidney at the age of 18 years [13]. Our patient is under long-term follow up in the Nephrology Department.

Conclusions: In children with positive family history of ADPKD screening ultrasonography of the kidney, performed at the request of the family, allows early diagnosis of sporadic present abnormalities of the kidney and urinary tract.
